# Tracking intracellular uptake and localisation of alkyne tagged fatty acids using Raman spectroscopy

**DOI:** 10.1016/j.saa.2018.01.064

**Published:** 2018-05-15

**Authors:** Lauren E. Jamieson, Jennifer Greaves, Jayde A. McLellan, Kevin R. Munro, Nicholas C.O. Tomkinson, Luke H. Chamberlain, Karen Faulds, Duncan Graham

**Affiliations:** a Centre for Molecular Nanometrology, WestCHEM, Department of Pure and Applied Chemistry, Technology and Innovation Centre, University of Strathclyde, 99 George Street, Glasgow G1 1RD, UK; b Strathclyde Institute of Pharmacy and Biomedical Science, University of Strathclyde, 161 Cathedral Street, Glasgow G4 0RE, UK; c WestCHEM, Department of Pure and Applied Chemistry, University of Strathclyde, 295 Cathedral Street, Glasgow G1 1XL, UK

**Keywords:** Spectroscopy, Raman, Lipids, Fatty acids, Alkyne tag, Intracellular imaging

## Abstract

Intracellular uptake, distribution and metabolism of lipids are tightly regulated characteristics in healthy cells. An analytical technique capable of understanding these characteristics with a high level of species specificity in a minimally invasive manner is highly desirable in order to understand better how these become disrupted during disease. In this study, the uptake and distribution of three different alkyne tagged fatty acids in single cells were monitored and compared, highlighting the ability of Raman spectroscopy combined with alkyne tags for better understanding of the fine details with regard to uptake, distribution and metabolism of very chemically specific lipid species. This indicates the promise of using Raman spectroscopy directly with alkyne tagged lipids for cellular studies as opposed to subsequently clicking of a fluorophore onto the alkyne for fluorescence imaging.

## Highlights

• Three different alkyne tagged fatty acids detected by Raman spectroscopy
• Relative intracellular uptake of each determined
• Intracellular localisation with subcellular resolution determined

## Graphical Abstract

**Figure:**
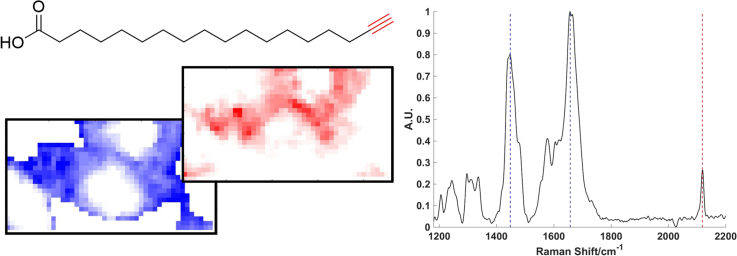

## 1 Introduction

Lipids are one of the four main classes of macromolecules. As such, they are important cellular components since tightly regulated uptake, distribution and metabolism of lipid species are vital for normal cell functioning. Dysregulation of these processes has been linked to disease states such as neurodegenerative disease [1] and cancer [2]. In order to develop new and effective treatment for such diseases it is important to gain a detailed understanding of underlying lipid biochemistry with respect to uptake, distribution and metabolism and changes during disease onset and progression. Better understanding will enable drug design for improved efficacy and therapies. In addition, one potential strategy for diagnostics and treatment monitoring is to detect divergence from the healthy lipid state and subsequent return through, for example, drug treatment by measuring a characteristic ‘snapshot’ of the state in healthy cells.

In order to gain insight into detailed aspects of lipid biochemistry and its regulation, it is vital to have analytical techniques capable of measuring these species. Lipids are a particularly understudied class of biomolecules due to the limitations in analytical techniques available to accurately image and characterize these species intracellularly. Many molecular biology based approaches for biochemical analysis require cells to be lysed and individual cell components isolated, such as the polymerase chain reaction, electrophoresis and Western blotting [3]. While these techniques can give a high level of chemically specific information, they are destructive and could introduce artefacts and modifications to the sample during the process of lysing and isolation of cell components. In addition, lipidomics is an emerging technique for lipid analysis but this is on a more global scale, requiring large sample quantities, which undergo extraction procedures, again making this a destructive technique [4]. Most conventional imaging strategies rely on fluorescently tagging biomolecules for detection of specific species [5]. This approach allows species to be imaged in their native environment non-destructively but there are disadvantages associated with the need for an external label to be added to the sample, which could itself interfere with the native state of the system. In particular, these techniques are less well established and limited in their ability to analyse lipids. Lipids lack inherent fluorescence, and the dyes available for staining lipid species tend to be very similar in size to the lipid species themselves, therefore having the potential to significantly alter innate distribution, dynamics and metabolism [6].

Raman spectroscopy is a promising technique for analysis of intracellular lipid species, due to the high Raman cross section associated with the largely non-polar lipid species giving rise to strong signals in the Raman spectra of these molecules [7]. While Raman scattering is a weak physical process, meaning measurements can be lengthy, it allows biological samples to be analysed in a label free manner with minimal sample preparation and non-destructively, allowing them to remain in their native state [8,9]. One of the major drawbacks when using Raman to analyse biological samples is that it is difficult to extract information about very specific species as opposed to a global response in, for example, lipid composition, due to the presence of broad peaks resulting from the overlapping of multiple peaks at slightly different frequencies [10]. Therefore, while this technique gives useful information regarding global distribution and dynamics of lipid species, it is difficult to assign signals to very specific lipids such as individual fatty acids.

In order to gain insight into lipid distribution and dynamics with regards to very specific biomolecules, a combination of a labelling approach with Raman imaging could be key. Alkyne tags have become important functional groups for labelling species for tracking using Raman spectroscopy. This utility stems from the unique alkyne signal observed in the biologically ‘silent’ region of the Raman spectrum between approximately 1800 and 2800 cm^−1^ [11]. Alkyne functionality is not an inherent characteristic of native biological systems and therefore in recent years a number of studies have created biorthogonal alkyne tagged reporters in order to image specific biological species or follow the dynamics of biological processes. Alkyne tags have also been incorporated into fluorescent imaging approaches, where they are added to biomolecules as precursors to later undergo click reactions with fluorescent tags typically introduced after cell fixation [12,13]. This approach has been used with alkynyl fatty acids previously to visualise lipid-modified proteins [13] using click chemistry-based detection and also more recently using a proximity-ligation strategy for detection of specific acylated protein [14]. The tags were introduced due to their small size, resulting in minimal interference with native biochemical processes, in comparison to traditional fluorescent imaging approaches. However, using Raman spectroscopy improves this technique further by removing the need for additional click reactions due to the ease at which the alkyne signal can be detected in the biological silent region of the Raman spectrum.

EdU (5-ethynyl-2′-deoxyuridine), a thymidine analogue with alkyne functionality, is one of the most studied alkyne tags for Raman tracking [12]. Tracking has moved from traditional Raman spectroscopy to the non-linear techniques of coherent anti-Stokes Raman and stimulated Raman spectroscopy to improve speed [[15], [16], [17], [18]]. Studies have incorporated alkyne modified fatty acids previously, however, these were primarily used as broad markers for lipids in comparison to proteins and nucleic acids, monitored using different alkyne tagged biomolecules [19,20]. To our knowledge, no previous work has directly compared the uptake and distribution of multiple different alkyne tagged fatty acids in a single study. In addition to the use of alkyne tagged biomolecules, deuterium tagging has been employed in a number of studies in an analogous approach [[21], [22], [23]], including comparison of uptake, metabolism and distribution of deuterated palmitic acid, oleic acid and cholesterol by macrophages [24]. However, in a comprehensive study, Yamakoshi et al. [11] demonstrated the superior capability of alkyne tagged compared to deuterium tagged molecules for Raman imaging studies. This study highlighted that although fully deuterated molecules could display relatively high Raman intensities with, for example, fully deuterated acetonitrile showing similar intensity for the nitrile compared to C—D stretch, when C—D bonds were not all identical the resultant signals were much more complex and individually lower in relative intensity. The study compared alkyne, nitrile, azide and fully deuterium tagged hexanoic acid, showing the alkyne tag to have the highest relative intensity for the alkyne stretching frequency, approximately 2.5× more intense than the nitrile equivalent, which from reference to acetonitrile had comparable signal intensity to incorporation of three identical C—D bonds. Therefore, alkyne tags have vast potential for probing uptake, distribution and metabolism of biomolecules, which has not yet been fully explored, particularly for comparing these characteristics for multiple structurally similar biomolecules.

## 2 Materials and Methods

### 2.1 Fatty Acids

Myristic acid, palmitic acid and stearic acid were purchased from Sigma Aldrich.

The synthesis of fatty acid alkyne probes is described in Greaves et al. [25].

### 2.2 Cell Culture

HEK293T cells were plated on glass coverslips in DMEM supplemented with 10% fetal bovine serum. For labelling with alkyne fatty acids, DMEM containing 1 mg/mL defatted BSA was added to the cells and the cells were incubated at 37 °C for 30 min. The medium was then removed and replaced with DMEM/BSA containing 100 μM of the fatty acid alkyne probe (C14, C16 or C18) and incubated for 4 h at 37 °C. Cells were then washed three times in PBS, fixed in 4% formaldehyde for 20 min, washed in PBS then H_2_O and air-dried overnight.

### 2.3 Raman Measurements

Raman spectra were acquired on a Renishaw inVia Raman microscope equipped with a 532 nm Nd:YAG laser giving a maximum power of 500 mW, 1800 lines/mm grating, and an Olympus 100×/NA 0.90 MPlanFL N objective.

For pure fatty acids and alkyne tagged fatty acids, a small sample of each was transferred onto a CaF_2_ window. Spectra of the solid crystals were acquired using 10 s acquisition time and 50% (*ca.* 10 mW) laser power for extended scanning from 200 cm^−1^ to 3200 cm^−1^. Three measurements were taken for each sample.

Fixed cells were mapped using a step size of 1 μm in x and y, 1 s acquisition time, 100% (*ca.* 20 mW*)* laser power and a spectral centre of 2000 cm^−1^. Three maps were acquired per condition.

### 2.4 Data Processing

#### 2.4.1 Pure Compounds

Spectra of pure compounds were processed using MATLAB® R2016a. Spectra were smoothed using Savitzky-Golay filtering with a polynomial order of 3 and a frame length of 9 followed by baseline subtraction using a custom script. Three spectra for each compound were min-max scaled and the average spectrum was calculated. For spectra of alkyne tagged fatty acids, the ratio of the intensity of the peak at 2110 cm^−1^ and the total spectral intensity between 1181 cm^−1^ and 2740 cm^−1^ was calculated for each spectrum.

#### 2.4.2 Cells

Cell maps were pre-processing using Renishaw Wire 4.2 software. Cosmic rays were removed using the nearest neighbour algorithm following by noise filtering and baseline subtraction. Pre-processed maps were then imported into MATLAB® R2016a. Cell regions were selected using a mask based on total spectral intensity to remove non-cell background. A false colour map of the intensity of the peak at 2118 cm^−1^ was created to map the presence of each alkyne in the cells. False colour images of the following peak intensity ratio for each cell were created:

• peak intensity 1448 cm^−1^/(peak intensity 1657 cm^−1^ + peak intensity 1448 cm^−1^).

The total intensity at 2118 cm^−1^ over each cell area was divided by the total spectral intensity over the respective cell area to give an indication of level of alkyne uptake and this was scaled based on the inherent intensity differences of the alkyne tagged fatty acids calculated previously.

A 2D median filter was used to create the images, where each pixel was the median value from the 2-by-2 neighborhood surrounding it.

## 3 Results and Discussion

**Fig. 1:**
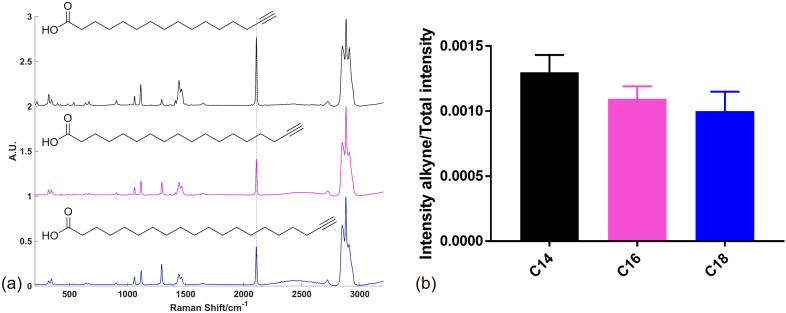
Alkyne tagged fatty acids. Average spectra of three alkyne tagged fatty acids and their structures (a) and relative intensity (mean + standard deviation) of the alkyne stretching frequency at 2110 cm^−1^ to the total spectral intensity between 1181 cm^−1^ and 2740 cm^−1^ for each tagged fatty acid (b). Black = C14 tag; pink = C16 tag; blue = C18 tag, dashed line in (a) indicates peak position of 2110 cm^−1^. Spectra were acquired using 532 nm excitation, 50%/*ca.* 10 mW laser power, 10 s acquisition time and an extended spectral range of 200 cm^−1^ to 3200 cm^−1^. Three spectra were acquired and min-max scaled for each compound and the average spectrum was plotted for each compound. Spectra were offset for clarity.

Three different alkyne tagged fatty acids were used in this study. These were designed to add an alkyne tag to myristic acid (C14), palmitic acid (C16) and stearic acid (C18) (Fig. 1). Initially spectra of each of these species were acquired to determine the chemically specific spectral signature associated with each compound, particularly the position and intensity of the carbon to carbon triple bond stretching frequency around 2100 cm^−1^ in the biologically silent region of the Raman spectrum. All compounds gave a strong Raman signal at 2110 cm^−1^ characteristic of the alkyne group C—C stretching mode (Fig. 1). In addition, a number of other characteristic peaks were observed in the lower wavenumber fingerprint region below *ca.* 1800 cm^−1^ and the higher wavenumber region from *ca.* 2800–3100 cm^−1^. From the spectra in Fig. 1(a), a visual difference in alkyne intensity relative to the rest of the peak intensities in the spectrum was apparent with C14 displaying a higher relative intensity compared to C16 and C18. In order to investigate this further, the intensity of the 2110 cm^−1^ alkyne signal was divided by the total spectral intensity for each spectrum between 1181 cm^−1^ and 2740 cm^−1^ (as this is the spectral range used for cell mapping centered at 2000 cm^−1^ subsequently) and the average was compared for three replicates of each species (Fig. 1(b)). This quantitative result supported the visual evidence that the spectral intensity of the alkyne signal compared to the total intensity in the selected spectral region was greater for C14 compared to C16 and C18, with mean values of 0.0013, 0.0011 and 0.0010 respectively. This information was important when quantifying relative uptake of each alkyne tag into cells, as the total alkyne signal intensity for a single cell (measured relative to the total spectral intensity) would therefore need to be scaled relative to the inherent compound to compound alkyne intensity differences. It was logical that the alkyne/total spectral intensity was highest for the C14 tag, followed by the C16 tag then the C18 tag as the alkyne group comprised a larger proportion of all chemical groups in the molecule *i.e.* C16 tag had an additional 2 × CH_2_ groups and the C18 tag had an additional 4 × CH_2_ groups.

**Fig. 2:**
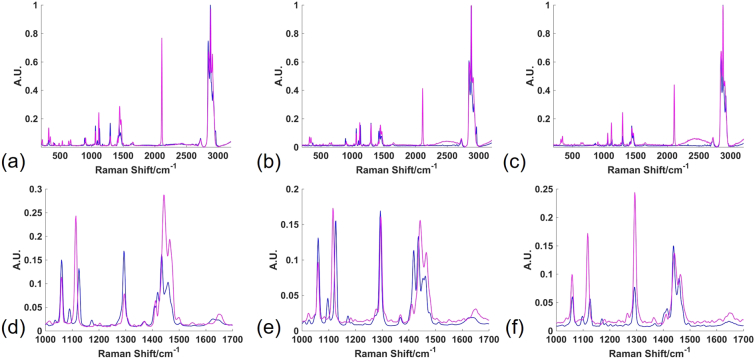
Tagged and untagged fatty acids. Average spectra of alkyne tagged and untagged C14 fatty acid (a); alkyne tagged and untagged C16 fatty acid (b); and tagged and untagged C18 fatty acid (c) in the full spectral range acquired between 200 cm^−1^ and 3200 cm^−1^. The spectral region between 1000 cm^−1^ and 1700 cm^−1^ for C14 (d), C16 (e) and C18 (f) alkyne tagged and untagged fatty acids is investigated more closely. Pink = alkyne tagged; blue = untagged. Spectra were acquired using 532 nm excitation, 50%/*ca.* 10 mW laser power, 10 s acquisition time and an extended spectral range of 200 cm^−1^ to 3200 cm^−1^. Three spectra were acquired and min-max scaled for each compound and the average spectrum was plotted for each compound.

In addition to investigating the spectra of the alkyne tagged fatty acids, these were compared to their counterpart untagged fatty acids — myristic acid, palmitic acid and stearic acid (Fig. 2). While relative spectral intensities were dependent on crystal orientation effects of the solid samples being measured, and varied acquisition to acquisition within a single sample, there were significant changes in peak presence (appearing or disappearing) and peak position (shifting) between untagged and tagged fatty acids. Assignments for the most significant of these are given in Table 1. Notably, the presence of the alkyne group was easily distinguished by the peak in the biologically silent region of the Raman spectrum at 2110 cm^−1^ in this case, but additional peaks at *ca.* 640 cm^−1^ and 670 cm^−1^ were also observed in the spectra of the tagged fatty acids, which were assigned to bending modes of the alkyne group [26]. Peaks in the region of the spectra between 1000 cm^−1^ and 1200 cm^−1^ were assigned to C—C stretching modes. This region displayed particularly interesting changes in untagged compared to tagged fatty acids. While the peak at 1060 cm^−1^ remained in the same position for all compounds, the peak at *ca.* 1095 cm^−1^ was only present in the untagged compounds and the peak at *ca.* 1125 cm^−1^ appeared at *ca.* 10 cm^−1^ lower in the tagged compared to untagged compounds. For untagged saturated fatty acids, the peak position at *ca.* 1095 cm^−1^ is known to reflect chain length, with an increase in chain length resulting in an increase in the Raman shift of this peak position [7]. For the tagged species investigated here, this peak was not present, however the peak at *ca.* 1115 cm^−1^ showed an increase in Raman shift with chain length from 1114 cm^−1^ to 1116 cm^−1^ to 1117 cm^−1^ for C14, C16 and C18 respectively. This was a shift of 11 cm^−1^, 11 cm^−1^ and 8 cm^−1^ lower than the respective untagged species. These observations suggested that the peak at *ca.* 1095 cm^−1^ was characteristic of a C—C stretch involving the CH_3_ group of the untagged species, and the shift to lower wavenumber of the *ca.* 1125 cm^−1^ peak in tagged species reflected a decrease in energy required for the C—C stretch vibration associated with an increase in the reduced mass and/or a decrease in the force constant associated with the bond. As the alkyne tag had a greater mass than the CH_3_ group it replaced, and was a more electron withdrawing group than the CH_3_ group it replaced, it was expected that both reduced mass increased and force constant of C—C bonds in the fatty acid chain, particularly those close to the alkyne tag, would decrease and thus the Raman shift would decrease.

**Table 1:**
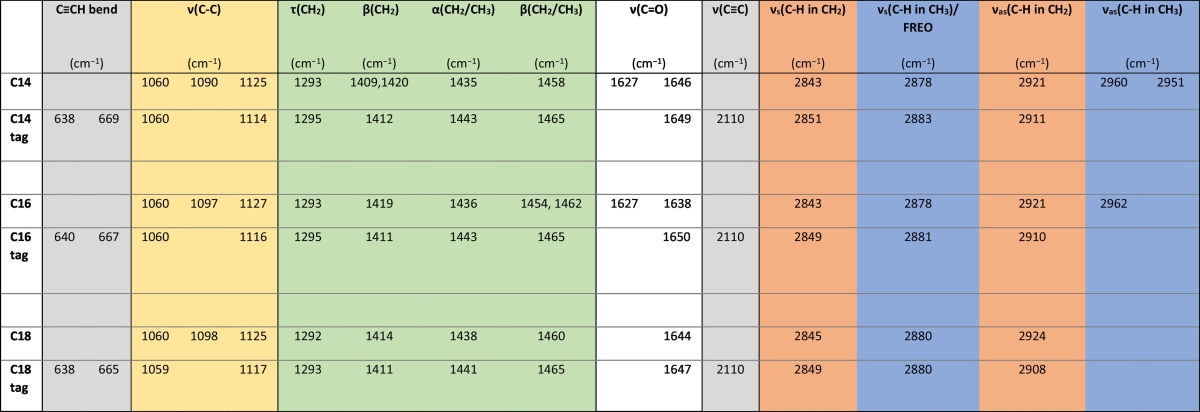
Fatty acid spectral assignments. Key assignments from Raman spectra of alkyne tagged and untagged C14, C16 and C18 fatty acids and their peak position for each species. Spectra were acquired using 532 nm laser excitation, 50%/*ca.* 10 mW laser power, 10 s acquisition time and an extended spectral range of 200 cm^−1^ to 3200 cm^−1^. Three spectra were acquired for each compound. The peaks are coded as follows: grey = alkyne group associated; yellow = C—C stretching; green = twisting, bending and scissoring of CH_2_ and CH_3_ groups; white = C=O stretch; orange = C—H stretch in CH_2_ groups; blue = C—H stretch in CH_3_ groups. *ν* = stretching (s = symmetric; as = asymmetric); τ = twisting; β = bending; α = scissoring; FREO = Fermi resonance enhanced overtone of CH_2_/CH_3_ deformation at *ca.* 1440 cm^−1^ [7,26,27,31].

In contrast, peaks associated with C—H twisting, bending and scissoring vibrations in CH_2_ and CH_3_ groups in the spectral region from *ca.* 1290 cm^−1^ to 1500 cm^−1^ primarily showed increases in Raman shift for the tagged compared to untagged species with the only exception being the bending mode at *ca.* 1415 cm^−1^. It is suggested that this bending mode corresponded to a C—H group closer to the alkyne tag/CH_3_, which thus responded more characteristically to the changing terminal group of the fatty acid. The vibrations that showed minimal or no change in Raman shift — particularly those at *ca.* 1295 and *ca.* 1060 cm^−1^ are suggested to originate further from the terminal end of the fatty acid chain, therefore showing less response to a change in terminal group. The C—H symmetric stretching and asymmetric stretching frequencies for CH_2_ groups at *ca.* 2850 cm^−1^ and 2920 cm^−1^ respectively appeared at higher and lower wavenumbers for the tagged compared to untagged species respectively, while the C—H symmetric stretching and asymmetric stretching frequencies for CH_3_ groups at *ca.* 2880 cm^−1^ and *ca.* 2960 cm^−1^ appeared at higher wavenumbers or not at all for tagged compared to untagged species. As the CH_3_ group was not present in the tagged species, it is likely that the C—H stretching peaks at *ca.* 2880 cm^−1^ for the tagged species actually originated from Fermi resonance enhanced overtones of the CH_2_ group deformations at *ca.* 1440 cm^−1^ [27], and as expected there was a loss of peaks in this region of the spectrum for the tagged species.

**Fig. 3:**
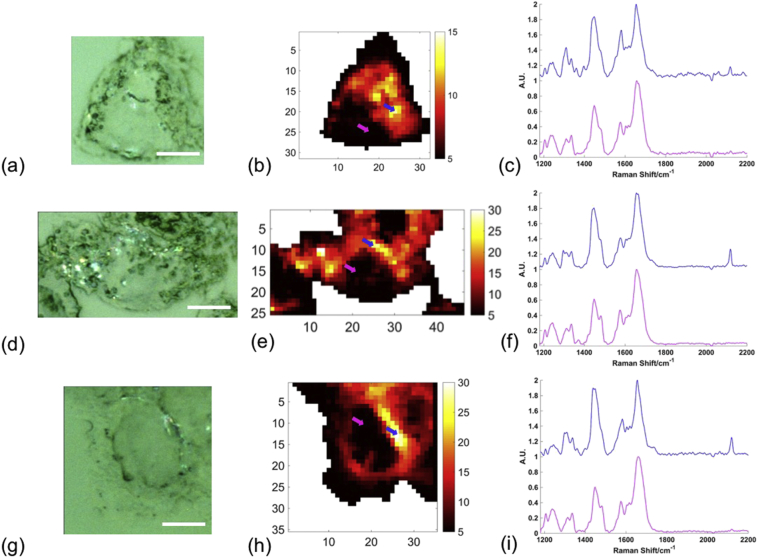
Intracellular uptake of alkyne tagged fatty acids. White light image of cell region mapped (a), false colour map of the same region showing the intensity of the 2118 cm^−1^ alkyne band (b) and examples of a high intensity spectrum (blue arrow in (b) and blue spectrum) and a low intensity spectrum (pink arrow in (b) and pink spectrum) (c) for HEK293T cells incubated for 4 h with 100 μM C14 alkyne tagged fatty acid. The same information is given for HEK293T cells incubated for 4 h with 100 μM C16 alkyne tagged fatty acid (d–f) and HEK293T cells incubated for 4 h with 100 μM C18 alkyne tagged fatty acid (g–i). Maps were acquired using a step size of 1 μm in x and y, 532 nm excitation, 100%/*ca.* 20 mW laser power, 1 s acquisition time and a spectral centre of 2000 cm^−1^. Scale bar = 20 μm. False colour image numbers on axis represent microns. Spectra were offset for clarity.

**Fig. 4:**
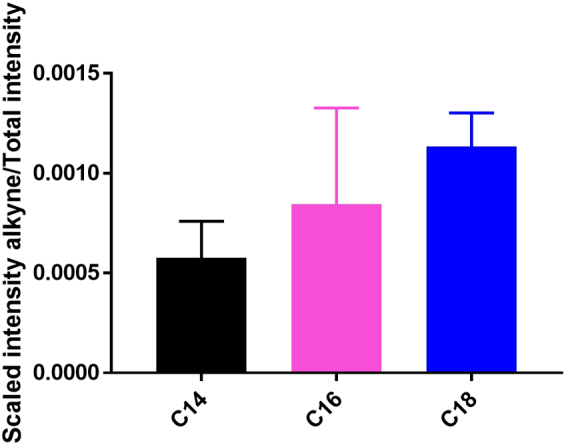
Relative uptake of alkyne tagged fatty acids. Relative intensity (mean + standard deviation) of the total sum of the intensity of the alkyne stretching frequency at 2118 cm^−1^ to the total spectral intensity for the total cellular area Raman mapped for HEK293T cells incubated for 4 h with 100 μM C14 alkyne, C16 alkyne or C18 alkyne, scaled to account for inherent intensity differences of the tags based on Fig. 1(b). Maps were acquired using a step size of 1 μm in x and y, 532 nm excitation, 100%/*ca.* 20 mW laser power, 1 s acquisition time and a spectral centre of 2000 cm^−1^. Three maps were acquired per tag. Black = C14 tag; pink = C16 tag; blue = C18 tag.

Each of the alkyne tags was added to HEK293T cells at a concentration of 100 μM for 4 h before fixing the cells for Raman analysis. Previous work from Greaves et al. [25] confirmed that these tags get taken up by HEK293T cells. Fixation with 4% paraformaldehyde is routinely employed in Raman studies of cells, and has been shown to minimally effect distribution of cellular biomolecules [28]. This protocol was followed in an analogous study using deuterated fatty acids [24]. Raman mapping was used to visualise and compare uptake of each of the tags to HEK293T cells (Fig. 3). These cells were used due to their high transfection efficiency to be exploited in future studies originating from this work. Cell regions were selected and mapped with a spectral centre of 2000 cm^−1^ (covering a spectral range of 1181 cm^−1^ to 2740 cm^−1^) using a 1 μm step size in the x and y direction. Fig. 3 shows an example map for each alkyne tag with the white light image showing the region that was mapped and the false colour image showing the intensity of the alkyne peak at 2118 cm^−1^. Example spectra from a region of high alkyne intensity and a region of no alkyne intensity are given, clearly showing the presence of an alkyne peak at 2118 cm^−1^ in the high intensity regions and very little or no signal in the low intensity regions. Interestingly, in this case the alkyne peak height in the high intensity examples is much higher relative to the rest of the spectrum for C16 and C18 than C14 suggesting significantly less C14 compared to C16 and C18 uptake. In addition, the inherent alkyne intensity for C14 has been shown to be relatively higher than the other species, therefore increasing this discrepancy further. Again, this was quantified by taking the ratio of the intensity of the alkyne stretching frequency divided by the total spectral intensity for three cells per alkyne tag (see Fig. S1 for images of alkyne distribution in additional two cells mapped per alkyne tag) and scaling based on the inherent signal intensity difference calculated in Fig. 1b (Fig. 4). These results quantify the previous observation, showing that there was a significantly lower uptake of C14 compared to C16 and C18 fatty acid with an average of 0.00058 compared to 0.00085 and 0.0011 respectively, equating to a 1.5× increase in uptake of C16 and a 1.9× increase in uptake of C18 compared to C14. While not completely quantitative, these estimates approximately agreed with those reported in Greaves et al. [25] using gas chromatography–mass spectrometry, where the relative uptake of C14 compared to C16 and C18 azide tagged equivalents showed a an increase compared to C14. This observation also falls in line with typical relative abundances of myristic acid, palmitic acid and stearic acid found intracellularly, with palmitic acid and stearic acid being two of the most predominant intracellular fatty acids [7,29]. It is powerful that Raman spectroscopy was able to predict relative uptake of these fatty acid tags in agreement with previous data, as it validates the accuracy of the technique but also enables the measurements to be made non-destructively, without the need for lyses and harsh extraction conditions, and in conjunction with subcellular localisation determination. Finally, the false colour maps showed clear distribution patterns of the alkynes inside cells, appearing to clearly surround a region thought to be the cell nucleus. In particular, for the cells incubated with C18, a clear intensity of alkyne signal was observed surrounding an oval region of no intensity thought to be the cell nucleus (Fig. 3(h)). As fatty acid synthesis and metabolism occur in the cytosol and mitochondria respectively, it is as expected that fatty acids remained outside of the cell nucleus.

**Fig. 5:**
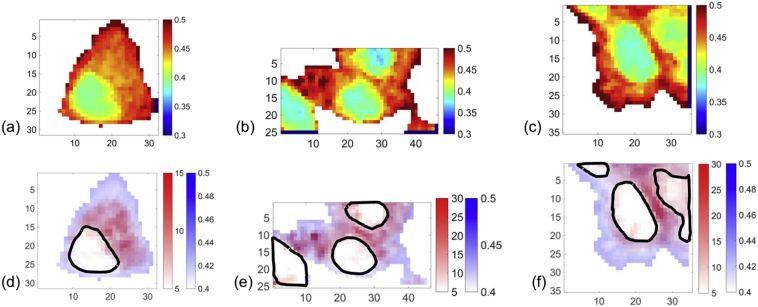
Intracellular localisation of alkyne tagged fatty acids. False colour images of the peak maximum intensity of the Raman signal at 1448 cm^−1^ divided by the sum of the peak intensities of the Raman signals at 1448 cm^−1^ and 1657 cm^−1^ for Raman maps of HEK293T cells incubated for 4 h with 100 μM C14 alkyne (a), for 4 h with 100 μM C16 alkyne (b) and for 4 h with 100 μM C18 alkyne (c). These images (blue) were overlaid with the respective false colour images of the intensity of the alkyne stretching peak at 2118 cm^−1^ (red) for C14 alkyne (d), C16 alkyne (e) and C18 alkyne (f) and regions attributed to nuclei were highlighted using black outline. Maps were acquired using a step size of 1 μm in x and y, 532 nm excitation, 100%/*ca.* 20 mW laser power, 1 s acquisition time and a spectral centre of 2000 cm^−1^. False colour image numbers on axis represent microns.

In order to further investigate intracellular localisation of the fatty acid tags, false colour mapping of the alkyne stretching frequency in the biologically silent region was combined with subcellular resolution images created using ratiometric analysis of further Raman peaks from cellular material in the fingerprint region of the spectrum. The ratiometric image generated from the intensity ratio of the peak intensity at 1448 cm^−1^ and the sum of the peak intensities at 1657 cm^−1^ and 1448 cm^−1^ (Fig. 5) indicated subcellular compartments (see Fig. S1 for analogous images for the further two cells mapped per alkyne tag). By dividing by the sum of these two maximum intensities, this ratio was also normalised between 0 and 1, with this approach rather than a simple ratio being demonstrated in previous studies also [30]. While both of these peaks have contributions from a number of biomolecular species present in the cell, the peak at 1448 cm^−1^ can be primarily associated with CH_2_ and CH_3_ group bending and scissoring vibrations, and due to the long aliphatic chains present in many lipid species, these contribute greatly to this region of the spectrum. The peak at 1657 cm^−1^ is strongly associated with amide I vibrations of proteins [10]. Therefore, this ratio approximately reflects the lipid/protein abundance across the cell and in particular highlights the nuclear regions with lower lipid/protein than cytoplasmic regions, reflective of the nuclear function to store DNA. In some images further subcellular compartments could be attributed to the higher intensity spots in the cytosolic region, potentially attributed to lipid droplets or even mitochondrial regions, where fatty acids are metabolised. Overlaying the alkyne image with the ratiometric lipid/protein image (Fig. 5(d–f)) provided further evidence regarding the subcellular localisation of the fatty acids taken up by cells with a similar distribution exhibited by all three fatty acids concentrated outside of the nuclear region. This appeared to be concentrated in the perinuclear region, for example for C18 tag (Fig. 5(f)), while in other cases the cytosolic distribution was more disperse throughout the cytoplasm with some high intensity spots, for example for C16 tag (Fig. 5(e)). The distribution in the perinuclear region is suggestive of potential localisation in the Golgi body and/or the endoplasmic reticulum, which are present in this area of the cell, and where high intensity spots were observed in cytosolic regions this could even indicate uptake into smaller organelles such as mitochondria or lipid droplets. The free fatty acids were initially complexed with bovine serum albumin to allow cellular uptake and it is thought that many of these subsequently become complexed to intracellular biomolecules. From previous work and the current study it is clear that alkyne tagged fatty acids were successfully taken up to HEK293T, allowing subsequent distribution and metabolism of these specific biomolecules to be monitored using Raman spectroscopy. By enabling incorporation of these fatty acids modified with small, and therefore unlikely to affect native biochemistry, alkyne groups into *in vitro* cell culture an incredibility useful platform for further studies monitoring changes to uptake, distribution and metabolism in response to, for example, disease or application of drugs with respect to specific fatty acids and how these can selectively change as a side effect or upon metabolic targeting in new therapeutic approaches. The foundation for this research has been established and will enable a greater understanding of biomolecule specific changes *in vitro* that will translate to a greater understanding of these processes *in vivo* and the development of more effective and selective drug candidates.

## 4 Conclusion

Lipids are an important class of biological macromolecules, which remain tightly regulated in the healthy cells, with onset of many diseases associated with dysregulation in uptake, distribution and metabolism of lipids. There are shortfalls in analytical techniques available to study lipid species intracellularly, and thus regulation, uptake, distribution and metabolism of lipid species still fails to be fully understood. The label free technique Raman spectroscopy holds many advantages when studying lipid species, but suffers from the downfall that it is difficult to pick out very specific species as opposed to a global lipid response. In this study the addition of a small alkyne tag to three different fatty acid species was used to study intracellular uptake and localisation of myristic, palmitic and stearic acid to HEK293T cells. To our knowledge this is the first time that this technique has been applied to study and compare the uptake and distribution of multiple different fatty acid species using alkyne tags and Raman spectroscopy. Relative quantification of level of uptake of the three different fatty acids studied was achieved and agreed with expected and previous results showing that myristic acid uptake was lower than that of palmitic and stearic acid. Furthermore, by combining information gained from monitoring the alkyne stretching frequency in the biologically silent region of the Raman spectrum with ratiometric imaging based on the fingerprint Raman region to identify subcellular compartments, an idea of subcellular localisation of each of the alkyne tags was gained. This approach holds great promise for understanding uptake, distribution and metabolism of lipids at a very species specific level in a minimally invasive manner, requiring only a small Raman active tag in the form of an alkyne group.

The following are the supplementary data related to this article.

Fig. S1 Intracellular uptake and localisation of alkyne tagged fatty acids. False colour images of cell regions mapped showing the intensity of the 2118 cm^−1^ alkyne band (a–c), and corresponding false colour images of the intensity of the Raman signal at 1448 cm^−1^ divided by the sum of the intensity of the Raman signals at 1448 cm^−1^ and 1657 cm^−1^ (d–f) for HEK293T cells incubated for 4 h with 100 μM C14 alkyne tagged fatty acid (a & d), HEK293T cells incubated for 4 h with 100 μM C16 alkyne tagged fatty acid (b & e), and HEK293T cells incubated for 4 h with 100 μM C18 alkyne tagged fatty acid (c & f). Maps were acquired using a step size of 1 μm in x and y, 532 nm excitation, 100%/*ca.* 20 mW laser power, 1 s acquisition time and a spectral centre of 2000 cm^−1^. False colour image numbers on axis represent microns. Image 1
