# Supplementary material for: Tracking intracellular uptake and localisation of alkyne tagged fatty acids using Raman spectroscopy
Source: Spectrochim Acta A Mol Biomol Spectrosc. 2018 May 15;197:30–6. doi: 10.1016/j.saa.2018.01.064 (PMC5890826; doi:10.1016/j.saa.2018.01.064)
Supplement: Fig. S1 — Intracellular uptake and localisation of alkyne tagged fatty acids. False colour images of cell regions mapped showing the intensity of the 2118 cm−1 alkyne band (a–c), and corresponding false colour images of the intensity of the Raman signal at 1448 cm−1 divided by the sum of the intensity of the Raman signals at 1448 cm−1 and 1657 cm−1 (d–f) for HEK293T cells incubated for 4 h with 100 μM C14 alkyne tagged fatty acid (a & d), HEK293T cells incubated for 4 h with 100 μM C16 alkyne tagged fatty acid (b & e), and HEK293T cells incubated for 4 h with 100 μM C18 alkyne tagged fatty acid (c & f). Maps were acquired using a step size of 1 μm in x and y, 532 nm excitation, 100%/ca. 20 mW laser power, 1 s acquisition time and a spectral centre of 2000 cm−1. False colour image numbers on axis represent microns. [file mmc1.docx]

**Electronic Supplementary Information**

**Tracking intracellular uptake and localisation of alkyne tagged fatty acids using Raman spectroscopy**

Lauren E. Jamieson^a^, Jennifer Greaves^b^, Jayde A. McLellan^c^, Kevin R. Munro^c^, Nicholas C.O. Tomkinson^c^, Luke H. Chamberlain^b^, Karen Faulds^a^, Duncan Graham^a*^

^a^Centre for Molecular Nanometrology, WestCHEM, Department of Pure and Applied Chemistry, Technology and Innovation Centre, University of Strathclyde, 99 George Street, Glasgow, G1 1RD, UK.

^b^Strathclyde Institute of Pharmacy and Biomedical Science, University of Strathclyde, 161 Cathedral Street, Glasgow, G4 0RE, UK.

^c^WestCHEM, Department of Pure and Applied Chemistry, University of Strathclyde, 295 Cathedral Street, Glasgow, G1 1XL, UK.

[*duncan.graham@strath.ac.uk](mailto:*duncan.graham@strath.ac.uk)


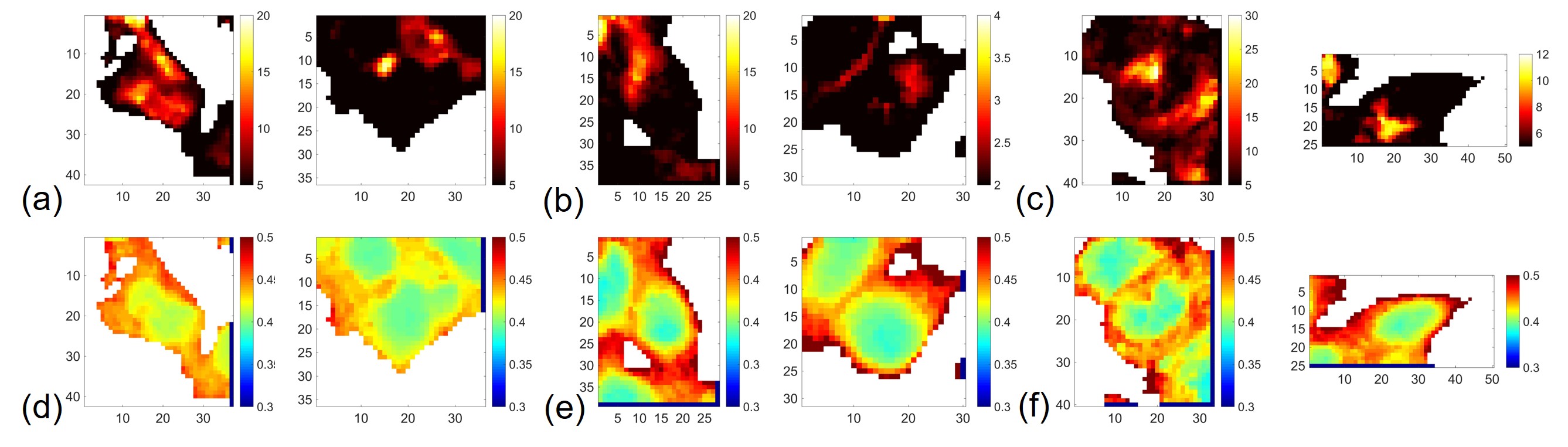


**Figure S1 Intracellular uptake and localisation of alkyne tagged fatty acids** False colour images of cell regions mapped showing the intensity of the 2118 cm^−1^ alkyne band (a-c), and corresponding false colour images of the intensity of the Raman signal at 1448 cm^−1^ divided by the sum of the intensity of the Raman signals at 1448 cm^−1^ and 1657 cm^−1^ (d-f) for HEK293T cells incubated for 4 hrs with 100 µM C14 alkyne tagged fatty acid (a&d), HEK293T cells incubated for 4 hrs with 100 µM C16 alkyne tagged fatty acid (b&e), and HEK293T cells incubated for 4 hrs with 100 µM C18 alkyne tagged fatty acid (c&f). Maps were acquired using a step size of 1 µm in x and y, 532 nm excitation, 100%/*ca.* 20 mW laser power, 1 s acquisition time and a spectral centre of 2000 cm^−1^. False colour image numbers on axis represent microns.
